# Dual action of the Gα_q_-PLCβ-PI(4,5)P_2_ pathway on TRPC1/4 and TRPC1/5 heterotetramers

**DOI:** 10.1038/s41598-018-30625-0

**Published:** 2018-08-14

**Authors:** Jongyun Myeong, Juyeon Ko, Misun Kwak, Jinsung Kim, Joohan Woo, Kotdaji Ha, Chansik Hong, Dongki Yang, Hyun Jin Kim, Ju-Hong Jeon, Insuk So

**Affiliations:** 1 Department of Physiology, Seoul National University College of Medicine, Seoul, 03080 Republic of Korea; 2 Department of Physiology and Biophysics, University of Washington School of Medicine, Seattle, WA 98195 USA; 3 Department of Physiology, Chosun University School of Medicine, Kwangju, 61452 Republic of Korea; 4 Department of Physiology, Gachon University College of Medicine, Incheon, 21936 Republic of Korea; 5 Department of Physiology, Sungkyunkwan University School of Medicine, Suwon, 16419 Republic of Korea

## Abstract

The transient receptor potential canonical (TRPC) 1 channel is widely distributed in mammalian cells and is involved in many physiological processes. TRPC1 is primarily considered a regulatory subunit that forms heterotetrameric channels with either TRPC4 or TRPC5 subunits. Here, we suggest that the regulation of TRPC1/4 and TRPC1/5 heterotetrameric channels by the Gα_q_-PLCβ pathway is self-limited and dynamically mediated by Gα_q_ and PI(4,5)P_2_. We provide evidence indicating that Gα_q_ protein directly interacts with either TRPC4 or TRPC5 of the heterotetrameric channels to permit activation. Simultaneously, Gα_q_-coupled PLCβ activation leads to the breakdown of PI(4,5)P_2_, which inhibits activity of TRPC1/4 and 1/5 channels.

## Introduction

The TRPC subfamily of the TRP channels consists of seven members. Among those, TRPC1, TRPC4, and TRPC5 channels are classified into a subgroup which can be activated by receptor stimulation^1^. TRPC4 and TRPC5 are expressed in selective tissues, including smooth muscle and neurons, and are non-selective cation channels which can be activated by specific G-protein alpha subunits^2,3^. TRPC1 is the first mammalian TRP channel to be cloned and is ubiquitously expressed in various tissues, but its membrane expression pattern and channel function remain controversial^4^.

Although homotetrameric TRPC1 is controversial about channel function, failure to regulate the expression or mutation of a TRPC1 channel is known to cause diseases. For example, the TRPC1 channel is upregulated in the squamous layer of the Darier’s disease patient^5^. It has been observed that tumor necrosis factor alpha exposure increases TRPC1 expression without significantly altering the expression of other TRPC isoforms in human pulmonary artery endothelial cells^6^. It is suggested that the TRPC1 channel is important for adaptation to biomechanical stress and that TRPC1 dysregulation can induce maladaptive cardiac hypertrophy and failure^7^. In TRPC1 knockdown neurons, specific migratory characteristics such as distance covered, locomotion speed, and directionality were increased^8^. In hippocampal neurons from *Trplc1/Trpc4/Trpc5*-triple-knockout (Trpc1/4/5^−/−^) mice, action potential-triggered excitatory postsynaptic currents were significantly reduced^9^.

Although TRPC1 is closely related to the diseases, its electrophysiological function is not observed. Since TRPC1 channel did not have electrophysiological function, most studies were concentrated on homotetrameric TRPC4 and TRPC5 channels. Recently, the current-voltage (I/V) relationships of the TRPC1/4 and TRPC1/5 channels exhibit an outward rectification, in contrast to the double rectifying TRPC4 and TRPC5 channels^8,10–12^. Thus, the TRPC1 channel has been suggested to be a regulatory subunit rather than a channel itself^12^.

Most TRP channels are regulated by signaling downstream of the Gαβγ timer, which hydrolyzes PI(4,5)P_2_^13–15^, generates second messenger lipids (IP_3_, DAG, and PKC), and increases the cytoplasmic Ca^2+^ concentration^16,17^, or by the direct binding of Gα^2,3,18^ and Gβγ subunits with channels^19^. For example, TRPC1^20,21^, TRPC3^14^, TRPC4^13^, TRPC5^21^, TRPC6^14,15,22,23^, TRPC7^14,15,23^, TRPV1^24^, TRPV3^25^, and TRPM8^26^ are regulated by phosphoinositides via GPCR stimulation. DAGs are stimulators of TRPC3, TRPC6, and TRPC7^27^. Desensitization of TRPC5 occurs via PKC phosphorylation^28^. TRPC4 is directly activated by Gα_i2_^2,3^, and TRPM8 is inhibited by interaction with Gα_q_^18^. Additionally, the TRPM1 and TRPM3 channels can be inhibited by Gα_o_^29^ or Gβγ^30,31^. It has been suggested that stimulation of Gα_q_-PLCβ-coupled receptors transiently activates TRPC1/4 and TRPC1/5 channels; however, the molecular mechanism of TRPC1/4 and TRPC1/5 activation by G protein signaling is largely unknown.

Here, we report a novel mechanism of self-limiting activation of TRPC1/4 and TRPC1/5 channels by G protein-coupled receptor (GPCR) stimulation. We used multiple assays to show that the heterotetrameric channels were activated by direct interaction with activated Gα_q_. Subsequently, activated channels underwent rundown due to the dissociation of PI(4,5)P_2_ from channel complexes by Gα_q_-dependent PI(4,5)P_2_ hydrolysis.

## Results

### Expression pattern and current function of homo- and hetero-tetrameric TRPC1α, TRPC4β, and TRPC5

**Figure 1:**
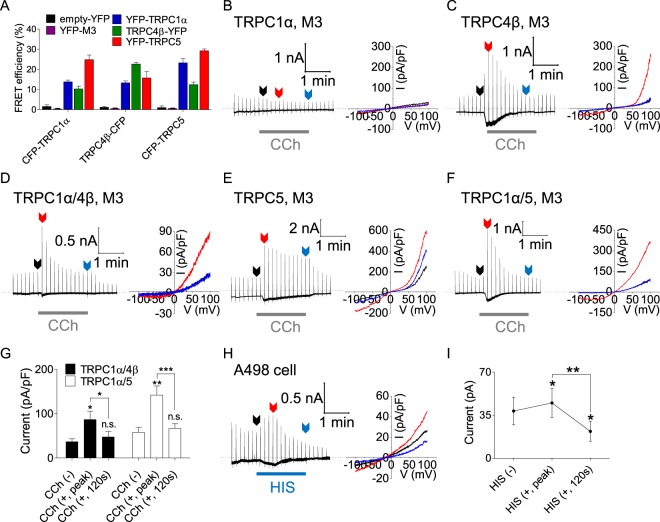
Properties of homo- and heterotetrameric TRPC1α, TRPC4β, and TRPC5 channels. **(A)** FRET efficiency between CFP- and YFP-tagged TRPC1α, TRPC4β, and TRPC5 channels, with empty YFP and YFP-M3 as negative controls. Full traces and I/V curves of homo- and heterotetrameric **(B)** TRPC1α, **(C)** TRPC4β, **(D)** TRPC1α/4β, **(E)** TRPC5, and **(F)** TRPC1α/5 following stimulation with 100 μM CCh. **(G)** Basal, peak, and desensitization currents of TRPC1α/4β (n = 9) and TRPC1α/5 (n = 10) with CCh treatment at +100 mV. **(H)** Renal carcinoma cell line A498 stimulated with 100 μM histamine. **(I)** Basal, peak value, and current magnitudes 2 minutes after stimulation were compared with the values prior to stimulation. All data are presented as the mean ± SEM. n.s., not significant, *p < 0.05, **p < 0.01, ***p < 0.001.

To study the regulation of TRPC4 and TRPC5 by TRPC1, we first investigated their expression pattern and observed the interactions between TRPC4β/4β, TRPC5/5, TRPC1α/4β, TRPC1α/5, and TRPC4β/5 pairs at the plasma membrane with Förster resonance energy transfer (FRET) (Fig. 1A). HEK293 cells were cotransfected with CFP- or YFP-tagged TRPC1 (CFP-TRPC1α and YFP-TRPC1α), TRPC4 (TRPC4β-YFP and TRPC4β-CFP) and TRPC5 (CFP-TRPC5 and YFP-TRPC5). As negative control experiments, we confirmed that FRET efficiency of CFP-tagged TRPC channels with cytosolic YFP (empty-YFP) or YFP-tagged muscarinic receptor 3 (YFP-M3) were almost zero FRET efficiency. A co-immunoprecipitation (Co-IP) assay provided additional evidence for the formation of heterotetrameric TRPC1α/4β, TRPC1α/5, and TRPC4β/5 (Supplementary Fig. S1). When coexpressed with TRPC4β, TRPC1α was successfully targeted to the plasma membrane, which was not the case when it was expressed alone^32^. TRPC1α affected the properties of both TRPC4β and TRPC5 currents. The I/V relationships of the heterotetrameric TRPC channels are substantially different from the doubly rectifying I/V shapes of homotetrameric TRPC4β or TRPC5 channels (Fig. 1C,E). The stimulation of M3 receptors with 100 μM carbachol (CCh) elicited outwardly rectifying currents in HEK293 cells expressing TRPC1α/4β (Fig. 1D) and TRPC1α/5 (Fig. 1F). A receptor-stimulated current was not observed when TRPC1α was expressed alone (Fig. 1B). These results suggest that TRPC1 contributes to the formation of a distinct pore in a complex with TRPC4β or TRPC5, although TRPC1 channel cannot function alone.

To exclude the possibility of heterotetrameric TRPC1α/4β or TRPC1α/5 channels mixed together with homotetrameric TRPC4β or TRPC5 channels in our coexpression system, we only recorded the currents from those cells in which the fluorescence intensity of YFP-TRPC1α was at least three times greater than that of TRPC4β-CFP or CFP-TRPC5 at the plasma membrane (Supplementary Fig. S2). When the fluorescence ratio of YFP-tagged TRPC1α to CFP-tagged TRPC4β or TRPC5 was at least 3, the I/V shapes of outwardly rectifying heterotetrameric TRPC1/4 and TRPC1/5 currents were recorded. However, when the fluorescence ratio of YFP to CFP was less than 3, double rectified or mixed currents were observed. Consequently, we only recorded whole-cell currents in cells with a fluorescence ratio of at least 3 and excluded the contribution of homotetrameric currents. Therefore, our experiments were limited to heterotetrameric TRPC1α/4β and TRPC1α/5 under these conditions.

### Inactivation of heterotetrameric channels after Gα_q_-PLC pathway activation

Following GPCR stimulation, TRPC1α/4β and TRPC1α/5 channels show transient activation (Fig. 1D,F,G)^10^. Single channel recording of TRPC1α/5 heteromeric channels showed a transient increase in open probability by CCh stimulation (Supplementary Fig. S3). Additionally, 100 μM histamine transiently activated currents with an outwardly rectifying I/V curve in the renal cancer A498 cells^33,34^, which express TRPC1 and TRPC4 (Fig. 1H,I). These results suggest that receptor agonists, such as histamine and acetylcholine, transiently activate heterotetrameric TRPC1/4 in native cells, as well as in HEK cells expressing TRPC1α/4β. Therefore, we first examined the molecular mechanism of channel inactivation after GPCR stimulation.

**Figure 2:**
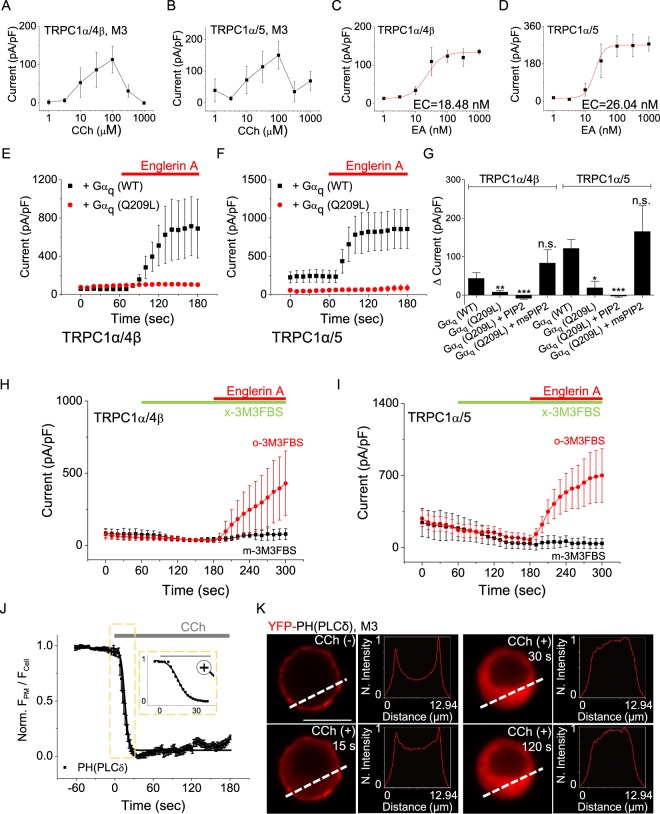
Inhibition of TRPC1α/4β and TRPC1α/5 by Gα_q_-PLCβ stimulation. The peak currents of **(A)** TRPC1α/4β and **(B)** TRP1α/5 were recorded using varying concentrations of CCh for stimulation. The magnitude of the **(C)** TRPC1α/4β **(D)** TRPC1α/5 currents due to EA stimulation was fitted to the Hill equation. Heterotetrameric **(E)** TRPC1α/4β and **(F)** TRPC1α/5 channels coexpressed with Gα_q_ (WT) or Gα_q_ (Q209L) were stimulated by 100 nM EA at +100 mV. **(G)** When PI(4,5)P_2_ or ms-PI(4,5)P_2_ was added or not to the pipette solution, delta currents (EA stimulated current*–*basal current) of cells expressing heterotetrameric channels and G alpha protein were quantified at 100 mV. Cells expressing **(H)** TRPC1α/4β or **(I)** TRPC1α/5 were pretreated with 100 μM m-3M3FBS or o-3M3FBS and stimulated with EA. **(J)** For cells expressing M3 and YFP-PH and stimulated with 100 μM CCh, the YFP-PH translocation curve was obtained by fitting the Norm data to a single-exponential decay function (black solid curve). The area enclosed by the dashed box is enlarged in the right panel. **(K)** The line scan shows the YFP-PH intensity along the white dashed line. All data are present as the mean ± SEM. n.s., not significant, *p < 0.05, **p < 0.01, ***p < 0.001. The scale bar represents 10 μm.

To understand how activated channels are inactivated over time by M3 receptor stimulation, we compared heterotetrameric channel activation dynamics using either the newly discovered direct and specific channel activator Englerin A (EA), which interacts with extracellular channel domains^33,34^ independently from GPCR mechanism, or CCh, a muscarinic receptor agonist. In HEK293 cells, the peak currents of TRPC1α/4β and TRPC1α/5 also increased in a concentration-dependent manner when up to 100 μM CCh was applied (Fig. 2A,B). Interestingly, when the concentration was greater than 100 μM, the magnitude of the peak current decreased. Unlike with CCh stimulation, activation was maintained without inactivation upon stimulation by 100 nM EA. The concentrations of EA required for 50% activation (EC_50_) of TRPC1α/4β and TRPC1α/5 were 18.5 nM (n = 6–8) and 26.0 nM (n = 6–8), respectively (Fig. 2C,D). The currents measured from TRPC1α/4β and TRPC1α/5 were slightly larger upon stimulation by 100 nM EA (TRPC1α/4β: 123.4 ± 16.1 pA/pF, n = 8; TRPC1α/5: 246.9 ± 49.2 pA/pF, n = 6) than after stimulation by 100 μM CCh (TRPC1α/4β: 112.7 ± 35.0 pA/pF, n = 6; TRPC1α/5: 150.2 ± 43.6 pA/pF, n = 7). Thus, we found that the activation properties of heterotetrameric channels were different upon stimulation by CCh than upon EA stimulation.

The main differences in the inactivation processes between CCh and EA were found in the GPCR downstream signaling pathway. To elucidate the role of Gα_q_-PLCβ downstream, we cotransfected HEK cells with TRPC1α/4β or TRPC1α/5 channels and wild-type Gα_q_ or the constitutively active Q209L mutant, which lacks intrinsic GTPase activity and, thus, mainly exists in the GTP-bound active conformation. Before doing these experiments, we confirmed the action of Gα_q_ on PLCβ downstream. We first measured plasma membrane PI(4,5)P_2_ hydrolysis directly by using a fluorescent indicator for PI(4,5)P_2_, which was the pleckstrin homology (PH) domain of phospholipase C δ1 (PLC-δ1) tagged with cyan or yellow fluorescent protein (CFP-PH or YFP-PH) (Supplementary Fig. S4). The PH domain of PLC-δ1 binds to both inositol 1,4,5-trisphosphate (IP_3_) and PI(4,5)P_2_ with high affinities^35^. K30, K32, R37, R38 and K57 of PLC-δ1 are required for interaction with PI(4,5)P_2_. As a control, CFP-PH^*^, which contains two point mutations in the phosphatidylinositol-binding pocket (Lys30 → Asn and Lys32 → Asn)^36^, was constructed. As expected, CFP-PH^*^ did not bind with PI(4,5)P_2_. The expression of the active mutant Gα_q_ (Q209L) activated PLCβ and, as a result, depleted plasma membrane PI(4,5)P_2_, whereas wild-type Gα_q_ did not affect. In the presence of wild-type Gα_q_, EA increased the current by 43.0 ± 15.2 pA/pF (n = 6) in cells expressing TRPC1α/4β (Fig. 2E,G). Notably, when Gα_q_ (Q209L) was coexpressed with TRPC1α/4β, EA failed to increase the current (by 7.6 ± 3.8 pA/pF, n = 7). In cells expressing TRPC1α/5, EA increased the currents of wild-type Gα_q_- and Gα_q_ (Q209L)-transfected cells by 121.0 ± 23.2 pA/pF (n = 7) and 18.6 ± 17.3 pA/pF (n = 6), respectively (Fig. 2F,G).

As an alternative method to deal with PI(4,5)P_2_ hydrolysis by PLCβ, we used pharmacological tools to activate PLCβ. As well known, the application of PLC activator m-3M3FBS (100 μM) induced PI(4,5)P_2_ hydrolysis and diacylglycerol (DAG) production as muscarinic stimulation, whereas there was no response to its inactive analog o-3M3FBS (Supplementary Fig. S5). To monitor DAG kinetics, we used YFP tagged DAG sensor (YFP-C1AC1A)^37^. After pretreatment of m-3M3FBS and o-3M3FBS, 100 nM EA was used to stimulate the TRPC1α/4β and TRPC1α/5 channels. With m-3M3FBS pretreatment to activate PLCβ, TRPC1α/4β and TRPC1α/5 currents were not activated by EA stimulation (Fig. 2H,I). However, during pretreatment with m-3M3FBS, the currents were slightly reduced. Unlike with m-3M3FBS, with o-3M3FBS pretreatment, TRPC1α/4β and TRPC1α/5 currents were increased by EA stimulation. Therefore, we suggest that the PLC downstream pathway is involved in the inactivation of heterotetrameric TRPC1α/4β and TRPC1α/5 channels but that the upstream pathway of PLCβ is not responsible for the inactivation of heterotetrameric channels.

Next, we postulated that decreasing the PI(4,5)P_2_ concentration would reduce the TRPC1α/4β and TRPC1α/5 currents. To confirm the effect of PI(4,5)P_2_ on the suppression of channel activity, we added PI(4,5)P_2_ directly to the internal solution of the pipette and tested whether added PI(4,5)P_2_ reverses the inactivation caused by the active mutant Gα_q_ (Q209L). In HEK293 cells coexpressing heterotetrameric channels and Gα_q_ (Q209L), the current increased for approximately 50 seconds after rupture and then decreased again (Supplementary Fig. S6A,C and Fig. 2G) when a pipette containing 20 μM of PI(4,5)P_2_ was used. The decreased current was not reactivated by EA. This phenomenon occurs because the added PI(4,5)P_2_ temporarily restores the activity of the channels, but the expressed Gα_q_(Q209L) protein hydrolyzes PI(4,5)P_2_. Thus, we used a metabolically stable form of PI(4,5)P_2_, diC8 ms-PIP2, to stably supply PI(4,5)P_2_, even in the presence of Gα_q_ (Q209L). When 20 μM diC8 ms-PI(4,5)P_2_ was added to the pipette solution, the current increased after rupture and increased further during EA stimulation (Supplementary Fig. S6B,D and Fig. 2G).

Similarly, activation of the M3 receptor depleted PI(4,5)P_2_ in HEK293 cells expressing M3 receptor (Fig. 2J,K). CCh stimulation also led to a decrease of PI(4,5)P_2_ and a subsequent increase of DAG even in HEK cells expressing TRPC1α/4β or TRPC1α/5 (Supplementary Fig. S7A,B). We compared the time-course between PI(4,5)P_2_ and DAG with the halftime for PI(4,5)P_2_ and DAG. The halftime constant (*Τ*_*1/2*_) for decreased PI(4,5)P_2_ level at the plasma membrane was smaller than that for the increased level of DAG at the plasma membrane. The *Τ*_*1/2*_ for the current inactivation of the heterotetrameric channel was the slowest among three half times. The *Τ*_*1/2*_ order of current >DAG >PIP2 was observed in all the concentrations (Supplementary Fig. S7C,D). Furthermore, as the concentration of CCh was higher, *Τ*_*1/2*_ was the faster. Based on these findings, we thought that PI(4,5)P_2_ depletion might be a candidate for the inactivation of TRPC1α/4β and TRPC1α/5 currents.

### PI(4,5)P_2_ is essential for maintaining TRPC1α/4β and TRPC1α/5 activation

To further establish the possible role of PI(4,5)P_2_ depletion during channel inactivation, we used two independent methods to deplete PI(4,5)P_2_ using two types of phosphatases, rapamycin inducible using Inp54p and VSP, while recording the channel activity. With these methods, we depleted PI(4,5)P_2_ only without generating IP_3_ or DAG.

**Figure 3:**
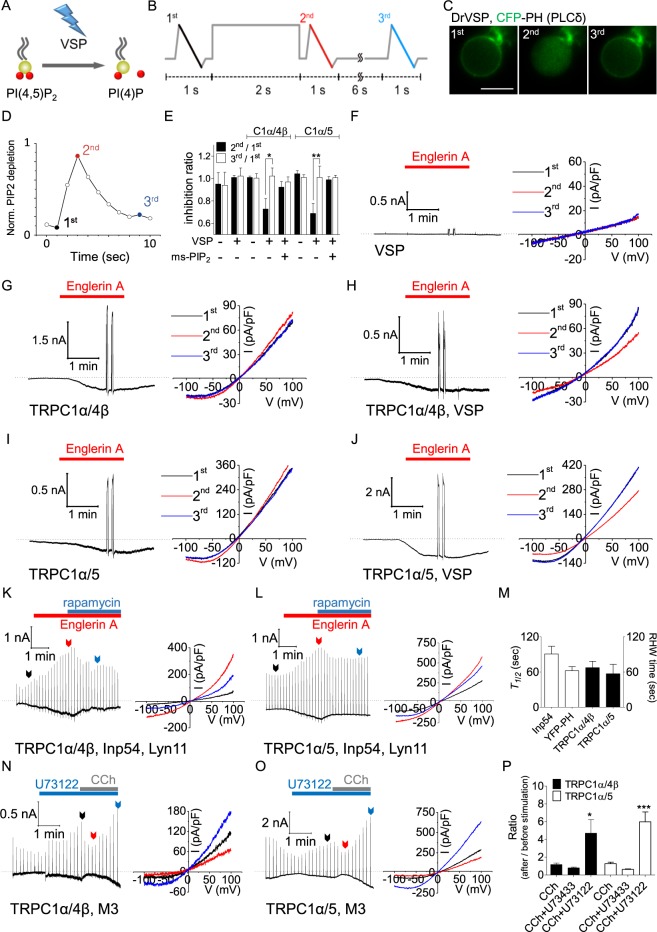
PI(4,5)P_2_ is necessary for maintaining TRPC1α/4β and TRPC1α/5 activity. **(A)** DrVSP is activated by depolarization and dephosphorylates PI(4,5)P_2_ to PI(4)P. **(B)** Cells were stimulated with 3TRP at +100 mV for 2 seconds before the 2^nd^ ramp. **(C,D)** PI(4,5)P_2_ dephosphorylation by DrVSP was observed during the 2^nd^ ramp pulse (n = 12, three independent experiments). The 2^nd^ ramp pulse stimulation was followed by a 3^rd^ ramp pulse stimulation after ~6 seconds. 3TRP applied to cells expressing **(F)** DrVSP, **(G)** TRPC1α/4β, **(H)** DrVSP and TRPC1α/4β, **(I)** TRPC1α/5, and **(J)** DrVSP and TRPC1α/5. **(E)** Summary of the inhibition ratio of the outward current for 3TRP at +100 mV in cells expressing VSP or with added ms-PI(4,5)P_2_. **(K,L)** The EA induction-activated TRPC1α/4β and TRPC1α/5 currents were inactivated by Inp54p and a 20 nM rapamycin perfusion. **(M)** Center values of sigmoidal curves (*Τ*_*1/2*_*)* depicting the CFP-FKBP-Inp54 and YFP-PH fluorescence intensities at the membrane and the RHW time of channel inhibition by rapamycin are presented as white and black bars, respectively. **(N)** TRPC1α/4β and **(O)** TRPC1α/5 currents increased continuously upon CCh perfusion and pretreatment with 20 μM U73122, which is a PLC inhibitor. **(P)** Summary of the reduction in the inactivation ratio for the TRPC1α/4β and TRPC1α/5 currents caused by U73122 and U73433 pretreatment. All data are presented as the mean ± SEM. *p < 0.05, **p < 0.01, ***p < 0.001; the scale bar represents 10 μm.

The first used expression of danio rerio voltage-sensing phosphatase (DrVSP), a membrane-resident voltage-controllable phosphoinositide phosphatase that dephosphorylates PI(4,5)P_2_ to phosphatidylinositol 4-phosphate (PI(4)P) (Fig. 3A), to reduce the endogenous level of PI(4,5)P_2_^38,39^. For this, we used the following three time ramp pulse (3TRP) protocol: the current was recorded at a 500-ms ramp from +100 to −100 mV with a holding potential of −60 mV; a brief step to +100 mV for 2 seconds to activate the phosphatase was followed by a 2^nd^ ramp pulse; the current was recorded by a 3^rd^ ramp pulse after ~6 seconds of recovery (Fig. 3B). Measurement of PI(4,5)P_2_ with CFP-PH showed PI(4,5)P_2_ depletion by the +100 mV pulse and recovery after ~6 seconds (Fig. 3C,D). In control experiments, only VSP transfected HEK293 cell currents (Fig. 3F) and the EA-activated TRPC1α/4β and TRPC1α/5 currents were the same at the 1^st^, 2^nd^, and 3^rd^ ramps in the absence of DrVSP (Fig. 3G,I). However, in the presence of DrVSP, depletion of PI(4,5)P_2_ reduced the TRPC1α/4β and TRPC1α/5 at 2^nd^ ramp current, respectively, relative to their original values (Fig. 3E,H,J). Addition of ms-PI(4,5)P_2_ to pipette solution prevented VSP induced currents depletion (Fig. 3E).

PI(4,5)P_2_ was also depleted using the FRB/FKBP rapamycin-inducible system. Supplementary Fig. 8A shows a schematic of the efficient depletion of PI(4,5)P_2_ by this system and the translocation of Inp54p-CFP from the cytosol to the membrane and of YFP-PH from the membrane to the cytosol. Treatment with 20 nM rapamycin recruited Inp54p to the plasma membrane, resulting in the time-dependent depletion of PI(4,5)P_2_ (Supplementary Fig. S8B,C). The depletion of PI(4,5)P_2_ with Inp54p reduced the EA-induced TRPC1α/4β and TRPC1α/5 currents (Fig. 3K,L). In a further experiment, the currents and movements of Inp54p and YFP-PH were simultaneously recorded. Interestingly, the tendency of EA-induced TRPC1α/4β and TRPC1α/5 currents to decrease was similar to the YFP-PH decrease in the cell membrane (Supplementary Fig. S8D,E). However, in the absence of the key protein, rapamycin stimulation did not decrease the TRPC1α/4β and TRPC1α/5 currents (Supplementary Fig. S8F,G). When ms-PI(4,5) was added to the pipette solution as CCh stimulation, rapamycin-induced Inp54p did not decrease the currents in heterotetrameric TRPC channels. We analyzed the kinetics of the PI(4,5)P_2_ decrease and the current decrease. Fitting the PI(4,5)P_2_ depletion time course to a sigmoidal curve revealed that both the PI(4,5)P_2_ depletion and the reduction in current had similar time courses, with *Τ*_*1/2*_ values for YFP-PH, TRPC1α/4β, and TRPC1α/5 of 62.2 ± 6.8 s, n = 7; 67.2 ± 10.9 s, n = 6; and 57.0 ± 16.1 s, n = 6, respectively (Fig. 3M). These results suggest that PI(4,5)P_2_ depletion without IP_3_ or DAG production induced the current inhibition of heterotetrameric channels.

To establish the relevance of PI(4,5)P_2_ hydrolysis for the inactivation observed during physiological GPCR stimulation, instead of the activator EA, we used the PLC inhibitor U73122, which completely inhibits the hydrolysis of PI(4,5)P_2_ during receptor stimulation (Supplementary Fig. S9A and B). Pretreating cells with U73122 gradually increased the currents and prevented time-dependent TRPC1α/4β and TRPC1α/5 current inactivation (Fig. 3N,O). In the presence of U73122, CCh still slightly reduced the current initially; however, importantly, CCh persistently increased the ratio (CCh stimulated current/ basal current) to 470% (TRPC1α/4β, n = 8) and 598% (TRPC1α/5, n = 7) (Fig. 3P). However, transient activation was observed during pretreatment with U73433, an inactive analog of U73122 (Supplementary Fig. S9C,D). Together, these results strongly suggest that PI(4,5)P_2_ regulates these channels activity and PI(4,5)P_2_ reduction decrease current of TRPC1α/4β and TRPC1α/5 by GPCR stimulation.

### Activated Gα_q_ directly binds and activates TRPC1α/4β and TRPC1α/5

**Figure 4:**
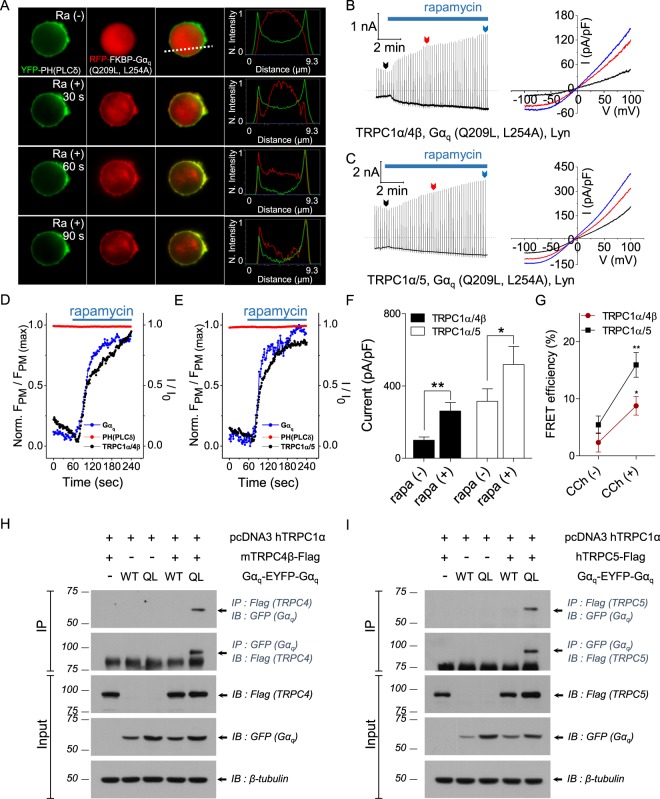
Activated Gα_q_ directly activates TRPC1α/4β and TRPC1α/5. **(A)** A cell coexpressing YFP-PH, RFP-FKBP-Gα_q_ (Q209L, L254A), and Lyn-FRB was stimulated by 20 nM rapamycin. The line scan shows the YFP-PH intensity along the white dashed line (4^th^ column). Images of before, 30 seconds after, 60 seconds after, and 90 seconds after the rapamycin perfusion are presented in separate rows. For cells expressing RFP-FKBP-Gα_q_ (Q209L, L254A) and Lyn-FRB with either heterotetrameric **(B)** TRPC1α/4β or **(C)** TRPC1α/5, the currents were recorded using a rapamycin-inducible system. Gα_q_ and PI(4,5)P_2_ indicator fluorescence motions and **(D)** TRPC1α/4β or **(E)** TRPC1α/5 current activity were measured simultaneously. Upon stimulation with rapamycin, the changes in current activity and the ratio of Gα_q_ and PI(4,5)P_2_ fluorescent indicators expressed at the cell membrane were normalized. **(F)** The current magnitudes of TRPC1α/4β and TRPC1α/5 before and after stimulation with rapamycin were measured. **(G)** Cells expressing Gα_q_ (WT)-YFP, M3, and TRPC1α with either TRPC4β-CFP or CFP-TRPC5 were stimulated by 100 μM CCh. Increases in the FRET efficiency of TRPC1α/4β and TRPC1α/5 with Gα_q_ (WT) are plotted. Mutual coprecipitation between **(H)** TRPC1α/4β and **(I)** TRPC1α/5 with mutants of Gα_q_. All data are presented as the mean ± SEM. *p < 0.05, **p < 0.01.

CCh stimulation activated TRPC1α/4β and TRPC1α/5 and continually increased the currents in the presence of a PLC inhibitor (Fig. 3N–P), and PLC activation did not induce an increase in heterotetrameric current (Fig. 2H,I). Because activation downstream of PLCβ is independent of channel activation, we postulated that Gα_q_ activates TRPC1α/4β and TRPC1α/5. To understand the activation mechanism of the heterotetramers, we employed the FRB/FKBP rapamycin-inducible system and Gα_q_ (Q209L, L254 A), which reportedly lacks the ability to activate PLCβ^40^. The induction of currents by rapamycin was not observed in the absence of key molecules (Supplementary Fig. S10). As shown in Fig. 4A, RFP-FKBP-Gα_q_ (Q209L, L254A) was translocated to the plasma membrane by 20 nM rapamycin treatment, and the mutant did not hydrolyze PI(4,5)P_2_. Importantly, the same treatment activated TRPC1α/4β and TRPC1α/5 heterotetramers (Fig. 4B,C,F), increasing the possibility that Gα_q_ interacted with and regulated the channels. In addition, the movements of PI(4,5)P_2_ and Gα_q_ were recorded simultaneously while recording currents from TRPC1α/4β and TRPC1α/5 (Fig. 4D,E). After normalization, TRPC1α/4β and TRPC1α/5 currents indeed showed similar dynamics to that of the normal translocation of RFP-FKBP-Gα_q_ (Q209L, L254A) to the plasma membrane.

To investigate the interactions between Gα_q_ and the heterotetrameric channels, we performed FRET measurements under receptor stimulation and co-IP experiments. The FRET efficiencies of TRPC1α/4β and TRPC1α/5 with Gα_q_ were initially 2.3 ± 1.7% and 5.4 ± 1.5% and increased to 8.8 ± 1.6% (n = 7) and 15.9 ± 2.2% (n = 7), respectively, after CCh stimulation (Fig. 4G). Co-IP analysis revealed that neither TRPC1α/4β nor TRPC1α/5 interacted with wild-type Gα_q_ but that both interacted with the activated Gα_q_ mutant (Fig. 4H,I). HEK293 cells expressing TRP1/4β-Flag (or TRPC1/5-Flag) and YFP-tagged Gα_q_ (WT) or Gα_q_ (Q209L) were lysed, and the lysates were immunoprecipitated by either a GFP antibody or a Flag antibody, and coprecipitation of TRPC4β (Fig. 4H) and TRPC5 (Fig. 4I) with Gα_q_ was detected with the indicated antibodies (top blots). Because receptor stimulation increased the membrane expression of the homotetrameric TRPC5 channel and increased the current^41^, we investigated whether CCh stimulation increased the cell membrane expression of the heterotetrameric TRPC1α/4β and TRPC1α/5 channels. When changes in the cell membrane expression were observed using a total internal reflection fluorescence (TIRF) microscope, the level of TRPC1α/4β expression did not significantly change with CCh stimulation (Supplementary Fig. S11A). However, in the case of TRPC1α/5, the expression level increased starting at 1 minute after CCh stimulation. In fact, because the transient activity of the current due to the CCh stimulation was a relatively rapid reaction, occurring within 30 seconds, it is difficult to understand how the increase in cell membrane expression caused the current increase. In addition, EA stimulation did not affect changes in the cell membrane expression of heteromeric channels (Supplementary Fig. S11B). In the surface biotinylation experiment, TRPC1α/4β did not react with CCh stimulation, but TRPC1α/5 showed an increase in surface expression 3 minutes after CCh stimulation (Supplementary Fig. S11C and D). Collectively, these results demonstrated that 1) activated Gα_q_ could activate TRPC1α/4β and TRPC1α/5 through direct binding to these channels and 2) PI(4,5)P_2_ depletion reduced the current of heterotetrameric channels.

### The Gα_q_-PLCβ pathway induces a biphasic response in TRPC1α/4β and TRPC1α/5 currents

**Figure 5:**
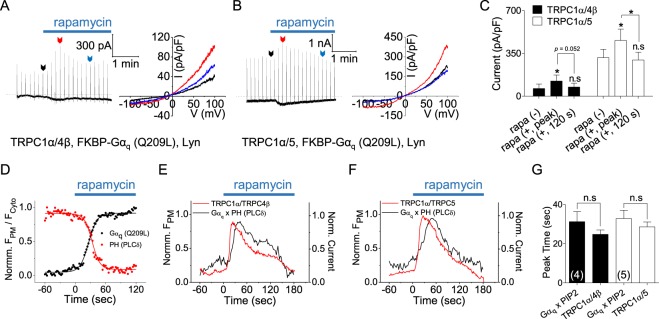
Biphasic regulation of TRPC1α/4β and TRPC1α/5 by Gα_q_/PI(4,5)P_2_. **(A)** TRPC4β-CFP or **(B)** CFP-TRPC5 was coexpressed with YFP-TRPC1α, RFP-FKBP-Gα_q_ (Q209L), and Lyn-FRB in HEK293 cells. Induction using 20 nM rapamycin led to biphasic current traces. **(C)** Basal, peak, and desensitization currents of TRPC1α/4β and TRPC1α/5 with rapamycin at +100 mV. **(D)** Rapamycin-induced curves of Norm. F_PM_*/*F_Cyto_ of Gα_q_ (black) and PH (red). **(E)** TRPC1α/4β and **(F)** TRPC1α/5 channel currents and Gα_q_ and PH fluorescence were simultaneously recorded. The curves of normalized current for TRPC1α/4β or TRPC1α/5 and the products of Gα_q_ and PH fluorescence are overlapped. **(G)** Bars represent the curve peak times for the multiplied Gα_q_ (Q209L) and PH curves and the current curves. All data are presented as the mean ± SEM; n.s., not significant. *p < 0.05.

Our results suggest that the transient activation of TRPC1α/4β and TRPC1α/5 by receptor stimulation consists of two components: direct activation by Gα_q_ and inactivation by PI(4,5)P_2_ depletion. To confirm this hypothesis, we reconstituted the components using the system shown in Fig. 4, except FKBP-Gα_q_(Q209L) was used instead of FKBP-Gα_q_(Q209L, L254A). As shown in Fig. 5A,B, rapamycin treatment transiently activated both TRPC1α/4β and TRPC1α/5, as observed in receptor stimulation. During rapamycin treatment, we observed the translocation of Gα_q_ to the plasma membrane and PI(4,5)P_2_ depletion (Fig. 5D). Heterotetrameric channel currents and the migration of Gα_q_ and PI(4,5)P_2_ were recorded simultaneously. Fig. 5E,F shows the currents and Gα_q_ x PI(4,5)P_2_ by rapamycin stimulation. We found that the peak times for the Gα_q_ x PI(4,5)P_2_ and for the currents were almost the same (TRPC1α/4β: Gα_q_ x PI(4,5)P_2_, 31.3 ± 5.2 s; current, 24.8 ± 2.2 s; n = 4; TRPC1α/5: Gα_q_ x PI(4,5)P_2_, 32.8 ± 4.3 s; current, 28.6 ± 2.5 s; n = 4) (Fig. 5E,F). When we induce Inp54 and Gα_q_(Q209L, L254A) to the membrane by rapamycin, TRPC1α/4β current shows transient activation (Supplementary Fig. S12). These results indicated that the transient activation of the heterotetramers in response to GPCR stimulation was due to activation by Gα_q_ and to inactivation by PI(4,5)P2 depletion.

## Discussion

We suggest here that the self-limiting regulation of TRPC1α/4β and TRPC1α/5 channels occurs through the Gα_q_-PLCβ pathway due to the following findings: (1) The Gα_q_ subunit binds directly to TRPC1α/4β and TRPC1α/5 and activates these channels. (2) A decrease in membrane PI(4,5)P_2_, caused by PLCβ activation, inhibits channel activity. The transient activation of heterotetrameric channels due to Gα_q_-PLC stimulation was observed not only in an overexpression system but also under general physiological conditions. Even when we activated TRPC1α/4β and TRPC1α/5 channels by EA, PI(4,5)P_2_ depletion with VSP or Inp54p inhibited the channels activity. PI(4,5)P_2_ was essential for the activation of TRPC1α/4β and TRPC1α/5 channels.

Stimulation of the renal cancer cell line A498 with EA results in an I/V curve with an outwardly rectifying shape for TRPC1/4, in contrast to the double rectifying shape observed for TRPC4α. We also recorded transient activation currents consistent with the I/V shape of TRPC1/4 after histamine stimulation in A498 cells (Fig. 1H). In endothelial cells, the nitrosylation of native TRPC1/5 heterotetrameric channels after G protein-coupled ATP receptor stimulation elicited entry of calcium^42^. Although the mechanism was different, the endogenous TRPC1/5 heterotetrameric channels in secretory fibroblast-like synoviocytes were activated by reduced thioredoxin^43^. In addition, stimulation of Q7 cells, a striatal cell line obtained from wild-type mice, showed an outwardly rectifying I/V shape for TRPC1/5, whereas in Q111 cells, a Huntington’s disease cell line obtained from mutant HTT knock-in mice, a doubly rectifying I/V shape was observed for TRPC5 because the expression of TRPC1 was suppressed^44^. Thus, studies on heterotetrameric TRPC1/4 or TRPC1/5 channels rather than homotetrameric TRPC4 or TRPC5 channels might have more pathophysiological relevance^8,43^.

Although TRPC1 does not form a functional homotetrameric ion channel, it is thought that the reason an abnormality in TRPC1 can cause disease is that the TRPC1 channel forms a heterotetrameric channel with TRPC4 and TRPC5. TRPC1 acts as a regulatory subunit that reduces the inward current of the homotetrameric TRPC4 and TRPC5 channels at negative potentials and increases the outward current at positive potentials, thus reducing cellular excitability. Single channel recording of the heterotetrameric channel showed reduced unitary conductance in TRPC1/4 and TRPC1/5 channels. Also, TRPC1 reduced the calcium permeability of TRPC4 or TRPC5 when forming heterotetramers^8,45^. Low levels of TRPC1 increased the formation of homotetrameric TRPC5, a highly Ca^2+^-permeable channel, and stimulated Ca^2+^-dependent apoptosis in Huntington’s disease cells^44^.

TRPC channels have been introduced as subunits of receptor-operated channels^46,47^ or store-operated channels^48,49^, although the precise mechanism by which the channel operates remains controversial. However, according to Fig. 2H,I, we can postulate that the TRPC1/4 and TRPC1/5 channels are not SOCs in HEK293 cells. In Fig. 2H,I, the PLC activator m-3M3FBS does not induce channel activity but instead appears to inhibit channel activity slightly. Pretreating cells with U73122 gradually increased the currents and prevented time-dependent inactivation of TRPC1α/4β and TRPC1α/5 currents by CCh stimulation (Fig. 3N,O,P). In addition, Gα_q_ (Q209L, L254A), non-hydrolyzing PI(4,5)P_2_ mutant form, activated TRPC1α/4β and TRPC1α/5 heterotetramers (Fig. 4B,C,F). On the contrary, in vascular smooth muscle cells, store depletion activated stromal interaction molecule 1 (STIM1) translocation to membrane where it formed STIM1-TRPC1 complexes to interact with Gα_q_ and PLCβ1^50,51^. Such an interaction induced by store depletion-activated TRPC1/5 heterotetramers in vascular smooth muscle cells. Recently Rubaiy *et al*.^52^ showed that pico145 is an excellent inhibitor of TRPC1/5 heterotetramer and pico145 did not inhibit SOC in HEK cells but inhibited TRPC1/5 channels expressed in HEK cells. In addition, Rubaiy *et al*. showed that pico145 did not inhibit SOC induced by TG in A498 cells and HUVEC. Whether TRPC1/5 is receptor-operated or store-operated depends upon the cellular context.

Molecular modeling predicted that the TRPV1 channel binds four PI(4,5)P_2_ molecules. When the structure of TRPV1 was determined^53^, the binding sites were revealed at the atomic level. These binding sites in TRPV1 (R409, R557, K571, R575, R579, K694) were responsible for the interaction with PI(4,5)P_2_, and similar sites have been identified in TRPC1α (H655), TRPC4β (K518, H630), and TRPC5 (K519, H634). In TRPC3, 6, and 7, channels bind PI(4,5)P_2_ with different sensitivities or affinities, but the binding sites were not determined^14,15^. In addition, other PI(4,5)P_2_ binding sites for other TRP channels have been proposed, such as the TRP domain of TRPM8^54^, K446 of TRPM1^55^ and the N terminus of TRPM4^56^. Therefore, the interaction sites of heterotetrameric TRPC1/4 and TRPC1/5 require more study. Additionally, we believe that the 3TRP protocol using VSP can be applied to other PI(4,5)P_2_-sensitive ion channels.

Interestingly, PI(4,5)P_2_ has been shown to be essential for TRPC4 activation^13^. Recently, it has been reported that PLCδ1, rather than PLCβ or PLCγ, is essential for the Gα_i_-mediated activity of the TRPC4 channel^16^. In fact, PLCδ1 is thought to play a central role in the regulation of the TRPC4 channel by PI(4,5)P_2_ and Ca^2+^. The concentrations of calcium, calmodulin, and PKC regulate TRPC4 and TRPC5^16,28,57–59^. Thus, studies on the complex interactions among PI(4,5)P_2_, PLCδ, PKC and calcium and their effects on TRPC1/4 and TRPC1/5 channels are needed.

It has been reported that the TRPC1 channel produces the TRPC1/3 heterotetramer, together with TRPC3, TRPC4, and TRPC5^8,60^. Therefore, it is necessary to study the mechanism of TRPC1/3 heterotetrameric channel activation and why no change in the I/V curve shape is observed, unlike with TRPC1/4 or TRPC1/5 heterotetramers, although TRPC1 decreases calcium permeability. Finally, the phenomenon that Gα_q_-PLC stimulation slowly increases the cell membrane expression of the TRPC1/5 channel (Supplementary Fig. S10A) also requires future study.

## Materials and Methods

### Cell culture and transient transfection

cDNA clones and human embryonic kidney (HEK293) cells (ATCC, Manassas, VA) were maintained according to the supplier’s recommendations. HEK293 cells were incubated in Dulbecco’s Modified Eagle’s Medium (DMEM) supplemented with 10% heat-inactivated FBS, penicillin (100 U/ml), and streptomycin (100 μg/ml) at 37 °C in a 5% CO_2_ humidified incubator. Cells were seeded in a confocal dish for recording images or a 12-well plate for whole-cell patch clamp recordings. The following day, XFP (CFP or YFP)-tagged channel and protein transfection was performed with Fugene-6 according to the manufacturer’s instructions. Electrophysiology or imaging experiments were performed the day after transfection.

### Electrophysiology

Cells were transferred onto a solution chamber on the stage of an inverted microscope (IX70, Olympus, Japan). Whole cell configuration was used to measure TRPC channel currents in HEK cells as described previously^2,44^. Cells were left for 10–15 min to attach to coverslips. Whole cell currents were recorded using an Axopatch 200B amplifier (Axon Instruments). Patch pipettes were made from borosilicate glass and had resistances of 3–5 MΩ when filled with normal intracellular solutions. The normal Tyrode (NT) contained 135 mM NaCl, 5 mM KCl, 2 mM CaCl_2_, 1 mM MgCl_2_, 10 mM glucose, and 10 mM HEPES with a pH that was adjusted to 7.4 using NaOH. The internal solution contained 140 mM CsCl, 10 mM HEPES, 0.2 mM Tris-guanosine 5′-triphospate, 0.5 mM EGTA, and 3 mM Mg-adenosine 5′-triphosphate with a pH that was adjusted to 7.3 with CsOH. A voltage ramp pulse from +100 mV to −100 mV was applied for 500 ms at a −60 mV holding potential. Experiments were performed at room temperature (18–22 °C). The recording chamber was continuously perfused at a flow rate of 1–2 ml/min.

### Microscopic image acquisition and FRET measurements

HEK293 cells were cultured in a 35-mm coverslip bottom dish or a 12-well plate to obtain images and measure FRET efficiency. To obtain the image and FRET efficiency of a cell, we used an inverted microscope with a 60x oil objective lens and the three-cube FRET calculation^61,62^ controlled by MetaMorph 7.6 (Molecular Devices, U.S.A). We mainly used three-cube FRET and mCherry (FF01-562/40, FF593-Di03, FF01-617/75, Semrock). The three-cube FRET efficiency (cube settings for CFP, YFP, and Raw FRET) was acquired from a pE-1 Main Unit to three-cube FRET (excitation, dichroic mirror, filter) through a fixed collimator: CFP (ET 435/20 nm, ET CFP/YFP/mCherry beam splitter, ET 470/24 nm, Chroma); YFP (ET 500/20 nm, ET CFP/YFP/mCherry beam splitter, ET 535/30 nm, Chroma); and CFP/YFP FRET (ET435/20 nm, ET CFP/YFP/mCherry beam splitter, ET535/30 nm, Chroma). The excitation LED and filter were sequentially rotated, the rotation period for each of the filter cubes was ~0.5 s, and all images (three for CFP/YFP/Raw FRET) were obtained within 2 s. Each of the images was acquired on a cooled 3 MHz (14 bit) EMCCD camera (iXon Ultra 888: ANDOR) with an exposure time of 100 ms with 1 × 1, 2 × 2, or 3 × 3 binning under the control of MetaMorph 7.6 software. Our FRET recording of the fluorophores was restricted in a range of CFP/YFP ratio from 0.5 to 2.0.

### FR and FRET efficiency computation

The FRET Ratio (FR)^62^ is equal to the fractional increase in YFP emission due to FRET and was calculated as *FR* = *F*_*AD*_*/F*_*A*_ = *[S*_*FRET*_*(DA*) *− R*_*D1*_ *· S*_*CFP*_*(DA)]/(R*_*A1*_ *· [S*_*YFP*_*(DA)–R*_*D2*_ *· S*_*CFP*_*(DA)])*. Here, *S*_*CUBE*_*(SPECIMENDA)* denotes an intensity measurement, where *CUBE* indicates the filter *cube (CFP, YFP, or FRET)*, and *SPECIMEN* indicates whether the cell is expressing the donor *(D; CFP)*, acceptor *(A; YFP)*, or both *(DA)*. *R*_*D1*_ = *S*_*FRET*_*(D)/S*_*CFP*_*(D), R*_*D2*_ = *S*_*YFP*_*(D)/S*_*CFP*_*(D)*, and *R*_*A1*_ = *S*_*FRET*_*(A)/S*_*YFP*_*(A)* are predetermined constants from measurements applied to single cells expressing only CFP- or YFP-tagged molecules. Although three-cube FRET does not require that CFP and YFP fusion constructs preserve the spectral features of the unattached fluorophores, similar ratios and recorded spectra furnished two indications that the spectral features of the fluorophores were largely unperturbed by fusion. Since the *FR* relies on YFP emission, YFP should be attached to the presumed limiting moiety in a given interaction. Subsequent quantitative calculations based on *FR* relied on a presumed 1:1 interaction stoichiometry. The effective FRET efficiency (*E*_*EFF*_) was determined by *E*_*EFF*_ = *E · A*_*b*_ = *(FR–1) · [E*_*YFP*_*(440)/E*_*CFP*_*(440)]*, where *E* is the intrinsic FRET efficiency when fluorophore-tagged molecules are associated with each other, *A*_*b*_ is the fraction of YFP-tagged molecules that are associated with CFP-tagged molecules, and the bracketed term is the ratio of YFP and CFP molar extinction coefficients scaled for the FRET cube excitation filter^63^. We determined this ratio to be 0.094 based on maximal extinction coefficients for ECFP and EYFP^64^ and excitation spectra.

### TIRF imaging

To conduct the plasma membrane-translocation assay of the channel using TIRF imaging, HEK293 cells were plated at 35-mm coverslip bottom dish. Cell were transfected with M3, TRPC1α and YFP tagged TRPC4β or TRPC5 using FuGENE 6 (Promega) transfection reagent according to manufacturer’s protocol. Imaging was performed at room temperature with a 100x oil objective on the stage of an IX81 TIRF microscope (Olympus, Tokyo, Japan). Fluorescence images of YFP were taken using a diode laser at 488 nm.

### Western blotting analyses, Co-IP, and surface biotinylation

For Western blotting, cells were seeded in 6-well plates. On the next day, 0.5–2 μg/well of cDNA was transfected into cells using the transfection reagent Lipofectamine 2000 (Invitrogen, U.S.A.) according to the manufacturer’s protocol. After transfection for 24 h, the cells were harvested as follows. Lysates were prepared in lysis buffer (0.5% Triton X-100, 50 Tris-Cl, 150 NaCl, 1 EDTA, pH 7.5, [in mM]) by being passed through a 26-gauge needle seven to 10 times after sonication. Lysates were centrifuged at 13,000 × *g* for 10 min at 4 °C, and the protein concentration in the supernatants was determined. The proteins extracted in sample buffer were loaded onto 8% Tris-glycine SDS-PAGE gels and then subsequently transferred onto a PVDF membrane. The proteins were probed with GFP (Invitrogen), Flag (Sigma), or β-Actin (GeneTex, U.S.A) antibodies for GFP-tagged, Flag-tagged, or housekeeping proteins as indicated.

### Surface biotinylation

PBS-washed cells were incubated in 0.5 mg/ml sulfo-NHS-LC-biotin (Pierce, U.S.A.) in PBS for 30 min on ice. Afterward, unreacted biotin was quenched by the addition of 100 mM glycine in PBS. The cells were then processed as described above to prepare cell extracts. Forty microliters of a 1:1 slurry of immobilized avidin beads (Pierce, U.S.A.) were added to 300 μl of the cell lysate containing 500 μg protein. After incubation for 1 h at room temperature, the beads were washed three times with 0.5% Triton-X-100 in PBS, and proteins were extracted in sample buffer. Collected proteins were then analyzed by Western blot.

### Statistical analysis

Data were analyzed using SPSS software (IBM SPSS Statistics 23). Results are given as mean ± SEM. Error bars indicate SEM. Here, *p < 0.05, **p < 0.01, and ***p < 0.001 were considered statistically significant, while n.s. indicates not significant. Results were compared using Student’s t-test. All data were generated from cells pooled from at least two biologically independent experiments. No samples were excluded.

## Electronic supplementary material

Supplementary Information
