# Supplementary material for: Dual action of the Gαq-PLCβ-PI(4,5)P2 pathway on TRPC1/4 and TRPC1/5 heterotetramers
Source: Sci Rep. 2018 Aug 14;8:12117. doi: 10.1038/s41598-018-30625-0 (PMC6092394; doi:10.1038/s41598-018-30625-0)
Supplement: Supplementary file 1 — Supplementary Information [file 41598_2018_30625_MOESM1_ESM.docx]

Supplementary information

Dual action of the Gα_q_-PLCβ-PI(4,5)P_2_ pathway on TRPC1/4 and TRPC1/5 heterotetramers

Jongyun Myeong^a,b,#^, Juyeon Ko^a,#^, Misun Kwak^a^, Jinsung Kim^a^, Joohan Woo^a^, Kotdaji Ha^a^, Chansik Hong^c^, Dongki Yang^d^, Hyun Jin Kim^e,*^, Ju-Hong Jeon^a^, and Insuk So^a,*^

^a^ Department of Physiology, Seoul National University College of Medicine, Seoul, 03080, Republic of Korea, ^b^ Department of Physiology and Biophysics, University of Washington School of Medicine, Seattle, WA, 98195, U.S.A, ^c^ Department of Physiology, Chosun University School of Medicine, Kwangju, 61452, Republic of Korea^, d^ Department of Physiology, Gachon University College of Medicine, Incheon, 21936, Republic of Korea, ^e^ Department of Physiology, Sungkyunkwan University School of Medicine, Suwon 16419, Republic of Korea

^#^ These authors contributed equally to this work.

* These authors are co-corresponding authors.

Corresponding authors

Insuk So, Department of Physiology and Institute of Dermatological Science, Seoul National University College of Medicine, Seoul, 03080, Republic of Korea, E-mail: insuk@snu.ac.kr Tel: +82-2-740-8228 Fax: +82-2-763-9667

Hyun Jin Kim, Department of Physiology, Sungkyunkwan University School of Medicine, Suwon 16419, Republic of Korea, E-mail: kimhyunjin@skku.edu

Supplementary Figures


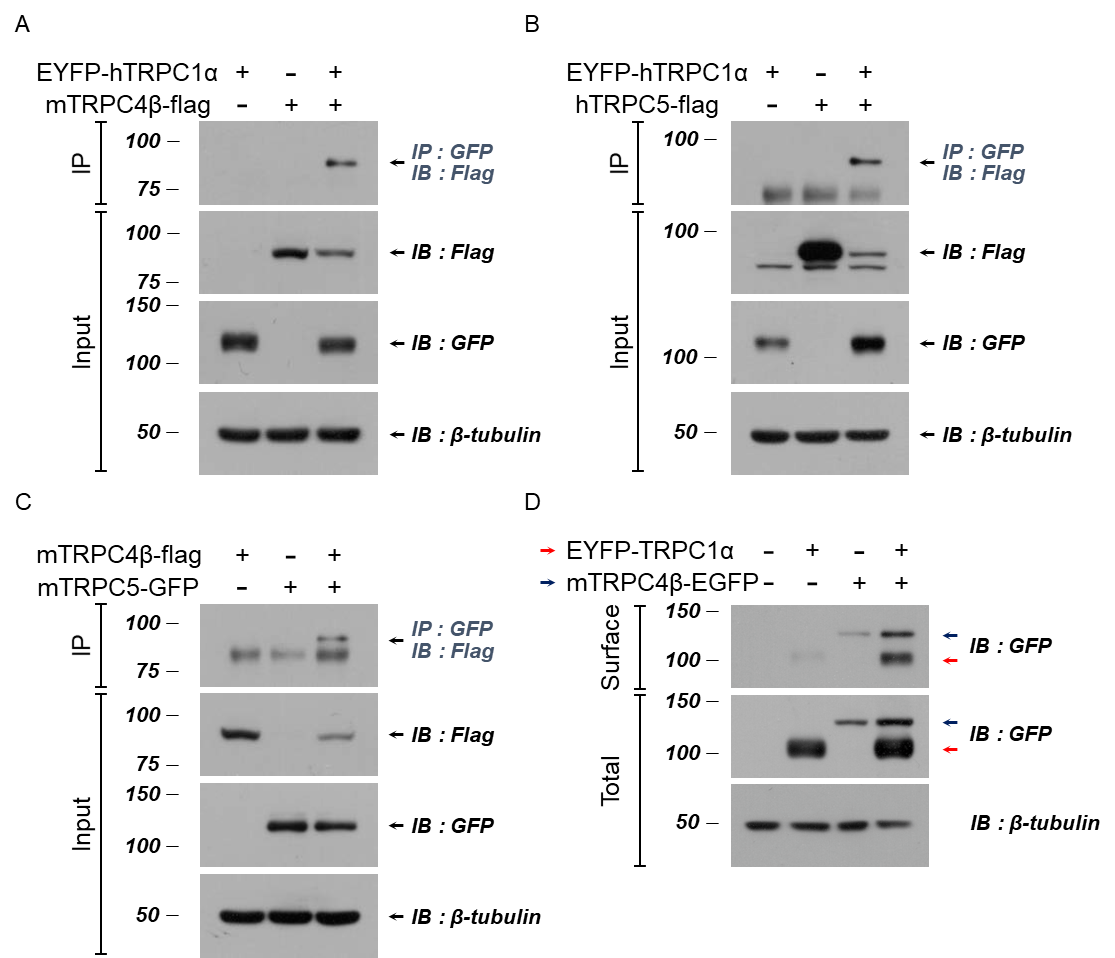


Supplementary Figure 1. TRPC1α, TRPC4β, and TRPC5 complexes and membrane expression of TRPC1α

Co-IP experiments for the (A) TRPC1α/4β, (B) TRPC1α/5, and (C) TRPC4β/5 heterotetrameric complexes. (D) Surface biotinylation in HEK293 cells expressing TRPC1α, TRPC4β, and TRPC1α+TRPC4β showed that TRPC1α has significantly lower surface levels than TRPC4β channels. The TRPC1α channel translocates to the plasma membrane when TRPC4β is coexpressed.


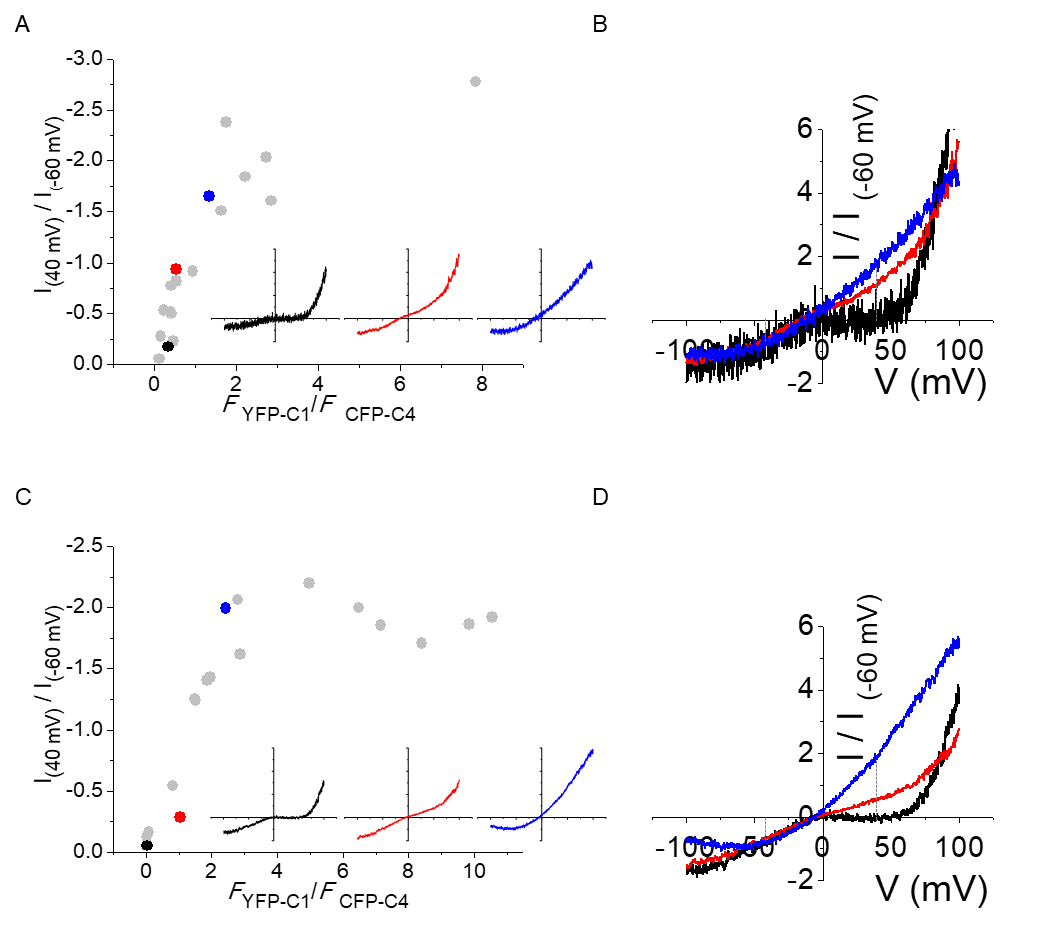


**Supplementary Figure 2. Comparison of I/V shape and intensity ratio of fluorescence-tagged proteins in a channel**

(A) The fluorescence intensity ratio of YFP/CFP in a cell expressing YFP-TRPC1α and TRPC4β-CFP is presented on the X-axis. Then, the current recorded at 40 mV in the cell was divided by the recorded current at -60 mV, and the value is presented on the Y-axis. (B) The I/V shape of the cells marked in black, red and blue. (C, D) In the same manner, cells expressing YFP-TRPC1 and CFP-TRPC5 were measured and recorded.


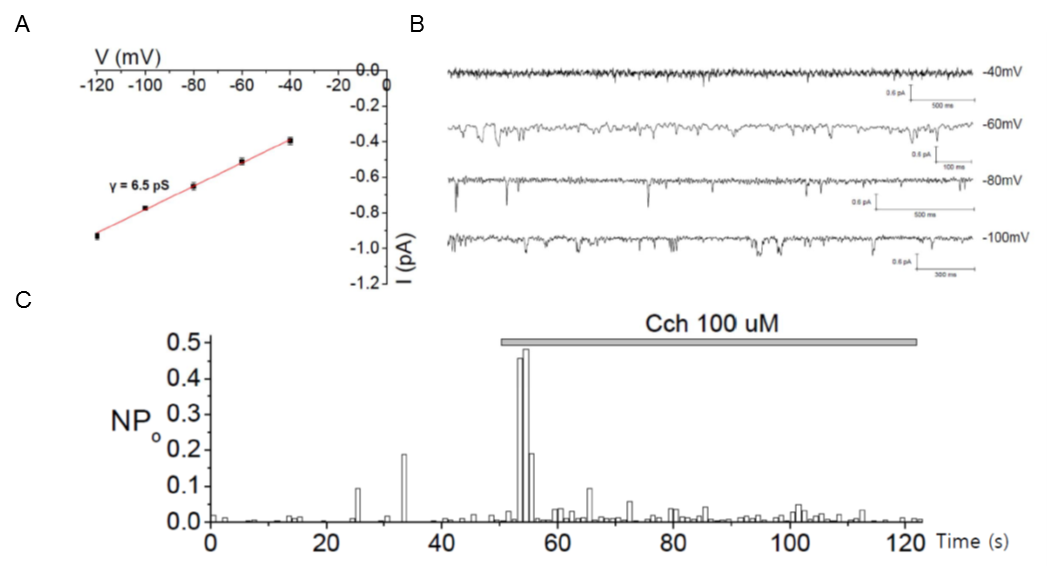


**Supplementary Figure 3. The single-channel recordings from the heterotetrameric TRPC1/5 channel.**

(A) The unitary conductance of heterotetrameric TRPC1α/5. (B) Raw current traces at the various membrane potentials. (C) NPo change during the application of CCh.


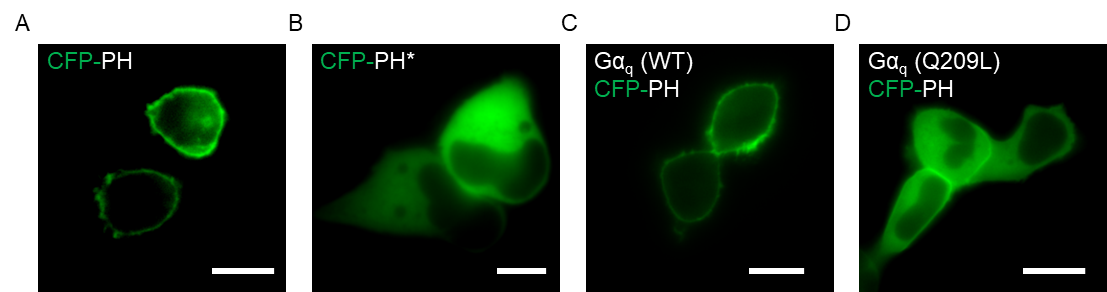


**Supplementary Figure 4. CFP-tagged PI(4,5)P_2_ biosensor (CFP-PH) distribution**

(A) Typical example images of CFP-PH in HEK293 cells. CFP-PH was predominantly localized to the plasma membrane. (B) The CFP-PH* (Lys30→Asn and Lys32→Asn) mutant was uniformly distributed in the cytosol. CFP-PH images in HEK293 cells cotransfected with (C) Gα_q_ (WT) or (D) Gα_q_ (Q209L). Scale bar, 10 μm.


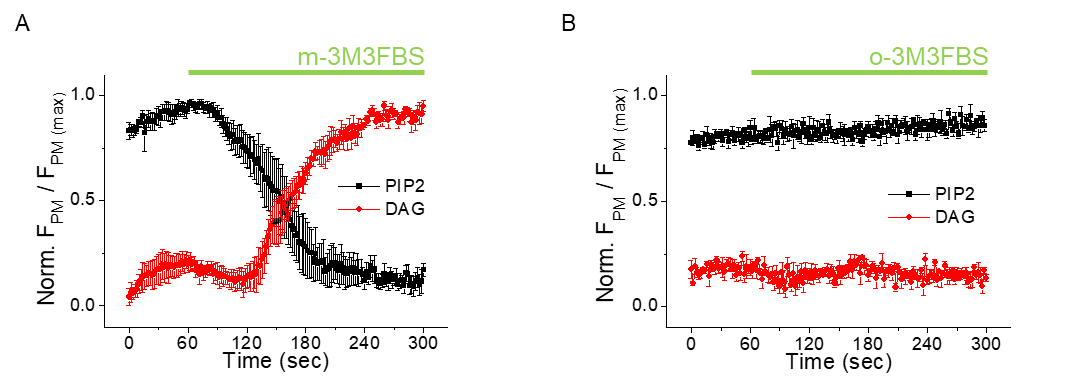


**Supplementary Figure 5. Movements of the PI(4,5)P­_2_ and DAG indicator after treatment with a PLC activator**

A 100 μM aliquot of (A) m-3M3FBS or (B) o-3M3FBS, an inactive analog of m-3M3FBS, was applied to investigate the role of PLC. Indicators of PI(4,5)P_2_ (black, CFP-PH) and DAG (red, YFP-C1AC1A) translocation were observed. All data are presented as the mean ± SEM.


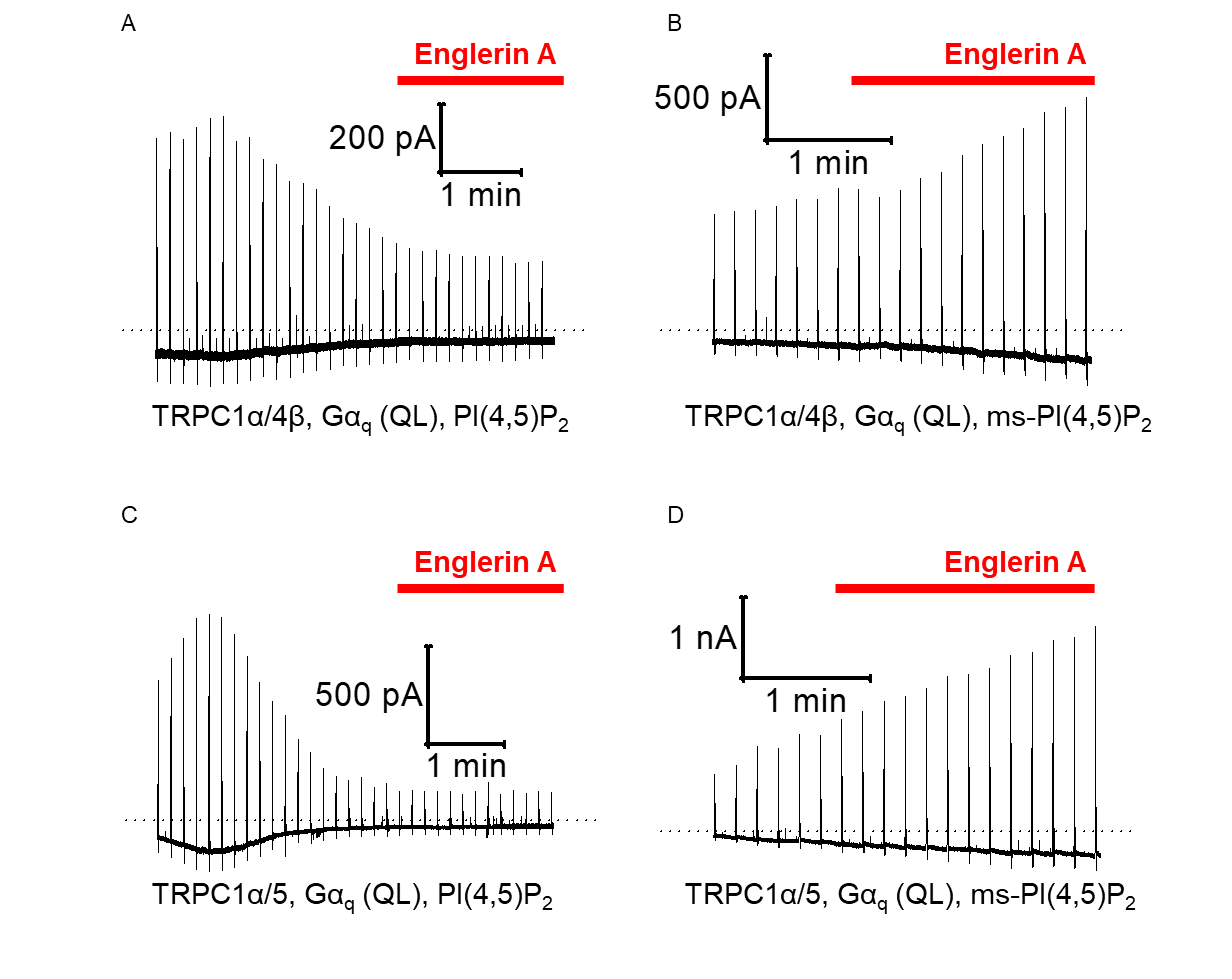


**Supplementary Figure 6. The activity of TRPC1α/4β and TRPC1α/5 when PI(4,5)P_2_ was added to the pipette solution.**

After the addition of 20 μM PI(4,5) to the pipette solution in a cell expressing (A) TRPC1α/4β and (C) TRPC1α/5 and Gα_q_ (QL), 100 nM EA was applied to stimulate the channels when the current became stable. Instead of PI(4,5)P_2_, the metabolically stable form of PI(4,5)P_2_, ms-PI(4,5)P_2_ dic8, was added to the pipette solution to record (B) TRPC1α/4β and (D) TRPC1α/5 currents.


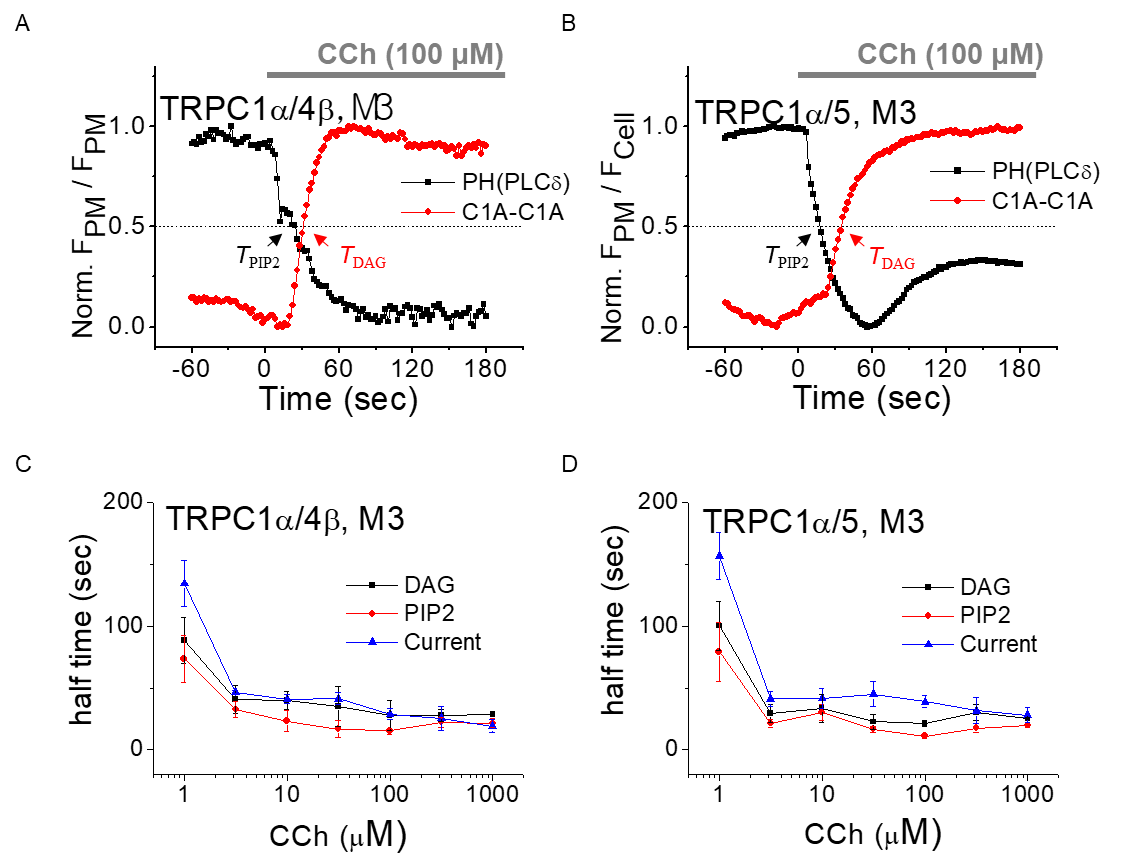


**Supplementary Figure 7. Kinetics of PI(4,5)_2_, DAG and currents after CCh stimulation.**

In cells coexpressing M3 and (A) TRPC1α/4β or (B) TRPCα1/5, the levels of change in PI(4,5)P_2_ (black, CFP-PH) and DAG (red, YFP-C1AC1A) concentrations were observed using indicators while applying 100 μM CCh. The fluorescence intensity ratio of membrane/cytosol indicator in a cell is presented on the Y-axis. The half membrane expression ratios of the PH domain and C1A-C1A are presented as *T_PIP2_* and *T_DAG._* The right half width (RHW) times of the (C) TRPC1α/4β and (D) TRPC1α/5 currents and the halftime of the DAG increase and the PI(4,5)P_2_ decrease were plotted against the CCh concentration. All data are presented as the mean ± SEM.


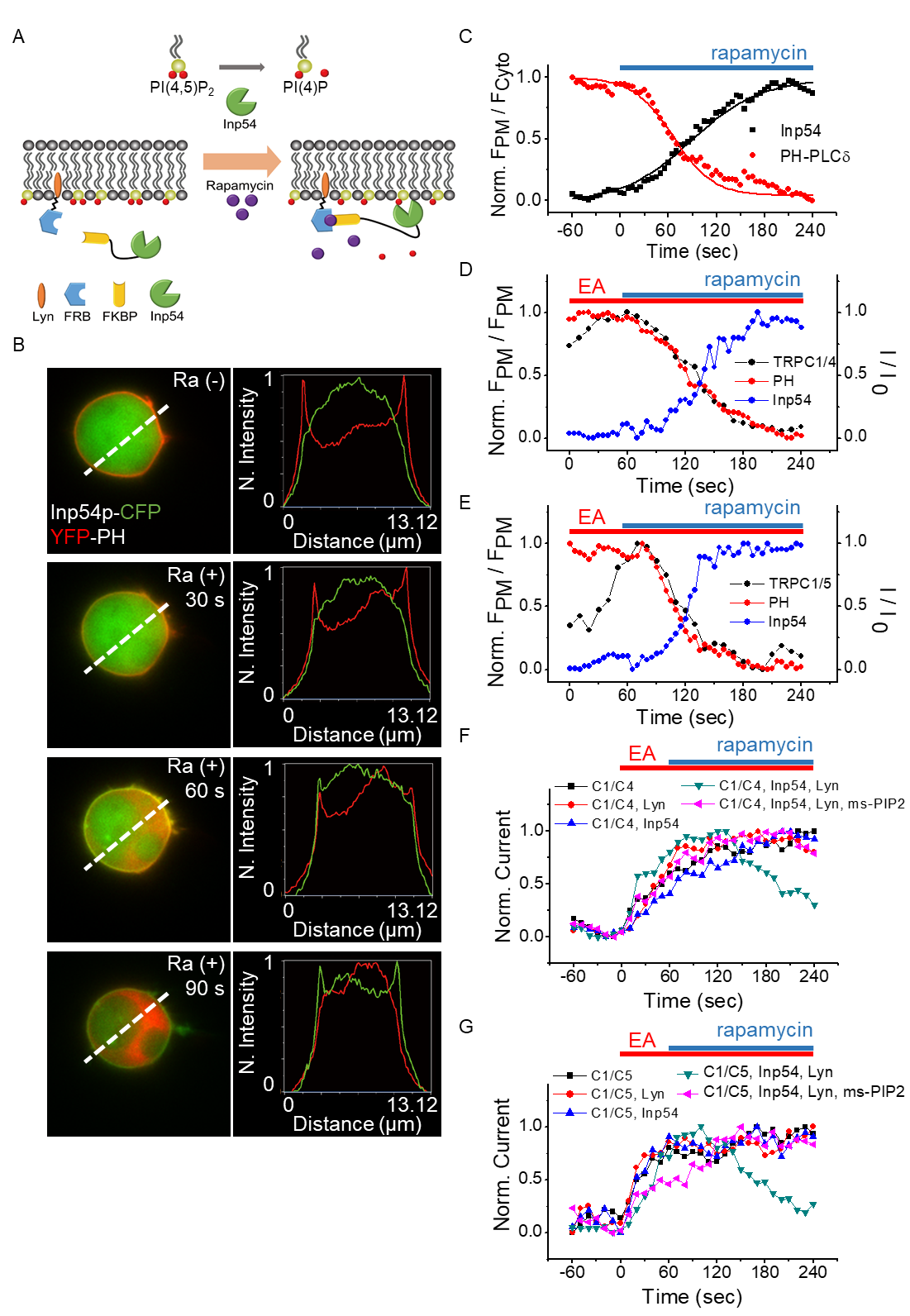


**Supplementary Figure 8. PI(4,5)P_2_ dynamics using the rapamycin-inducible system and the PLC inhibitor**

(A) Schematic representation of the PI(4,5)P_2_ depletion system. The addition of rapamycin induces the heterodimerization of the FRB and FKBP domains in Lyn-FRB and CFP-FKBP-Inp54p and thus causes the translocation of CFP-FKBP-Inp54 to the plasma membrane, where it degrades PI(4,5)P_2_. (B, C) Rapamycin at 20 nM induces the rapid translocation of CFP-FKBP-Inp54 (green) from the cytosol to the plasma membrane and the dissociation of the PI(4,5)P_2_ biosensor (YFP-PH, red) from the plasma membrane. In cells expressing PH, Inp54p, Lyn and (D) TRPC1α/4β or (E) TRPC1α/5, the current change and movement of PH and Inp54p fluorescence were simultaneously recorded. In cells expressing Lyn, Inp54p, (F) TRPC1α/4β or (G) TRPC1α/5 or in the absence of key molecules or with added ms-PI(4,5)P_2_, the current recorded by stimulation with 20 nM rapamycin and 100 nM EA was normalized.


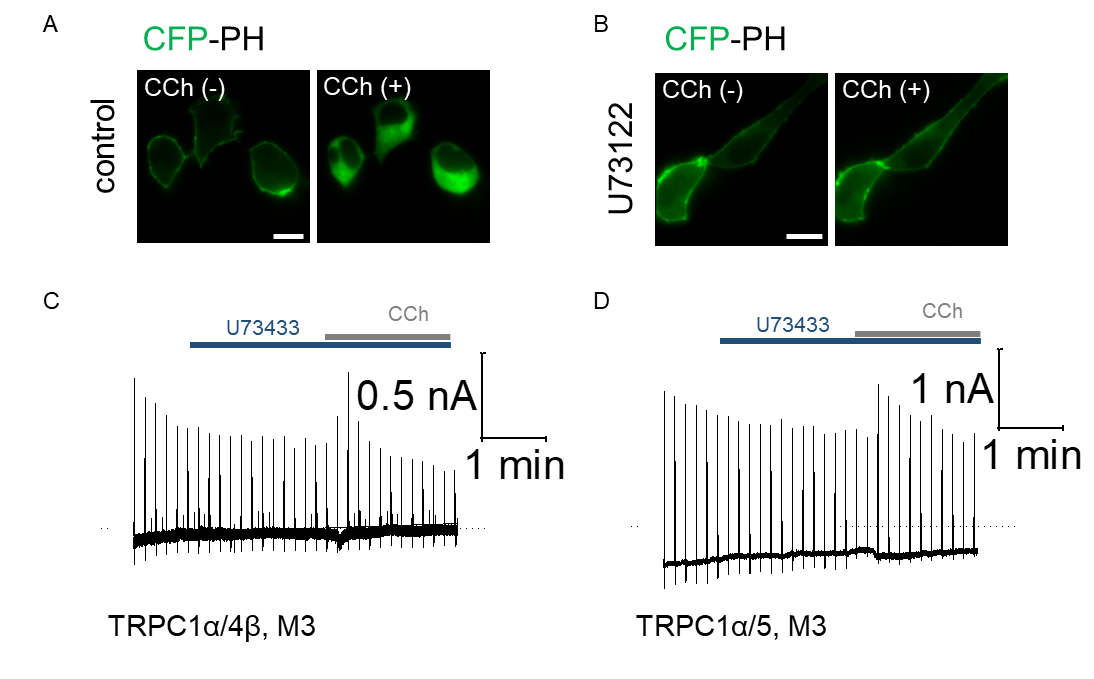


**Supplementary Figure 9. CCh stimulation and PLC blocker**

(A) When CCh was added at 10 μM, CFP-PH translocation was observed. (B) Pretreatment with 20 μM U73122 completely blocked the CFP-PH translocation. Cells expressing M3 and (C) TRPC1α/4β or (D) TRPC1α/5 were pretreated with 20 μM U73433, followed by 100 μM CCh stimulation. Scale bar, 10 μM.


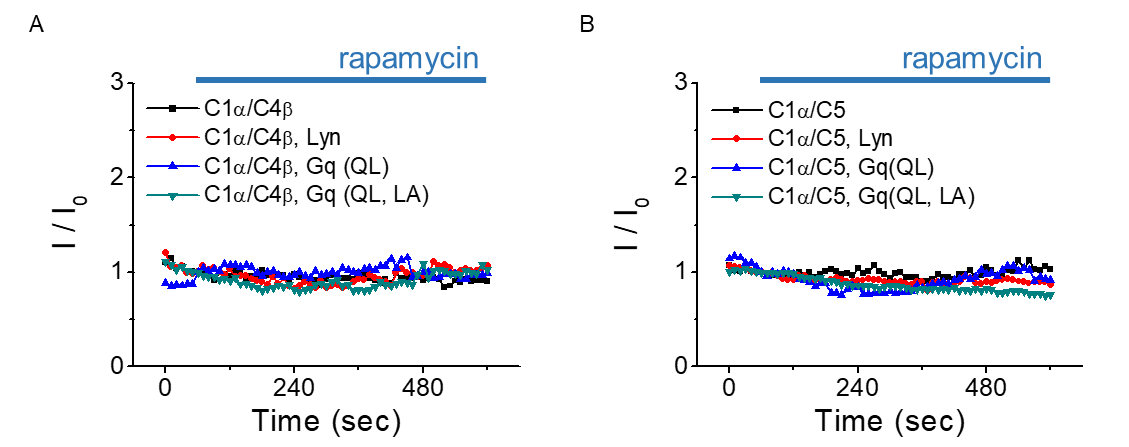


**Supplementary Figure 10. Control experiments using a rapamycin-inducible system.**

Negative control experiments using a rapamycin-inducible system. Lyn-FRB, RFP-FKBP-Gα_q_, (A) TRPC1α/4β or (B) TRPC1α/5 stimulated by 20 nM rapamycin in cells excluded from key molecules.


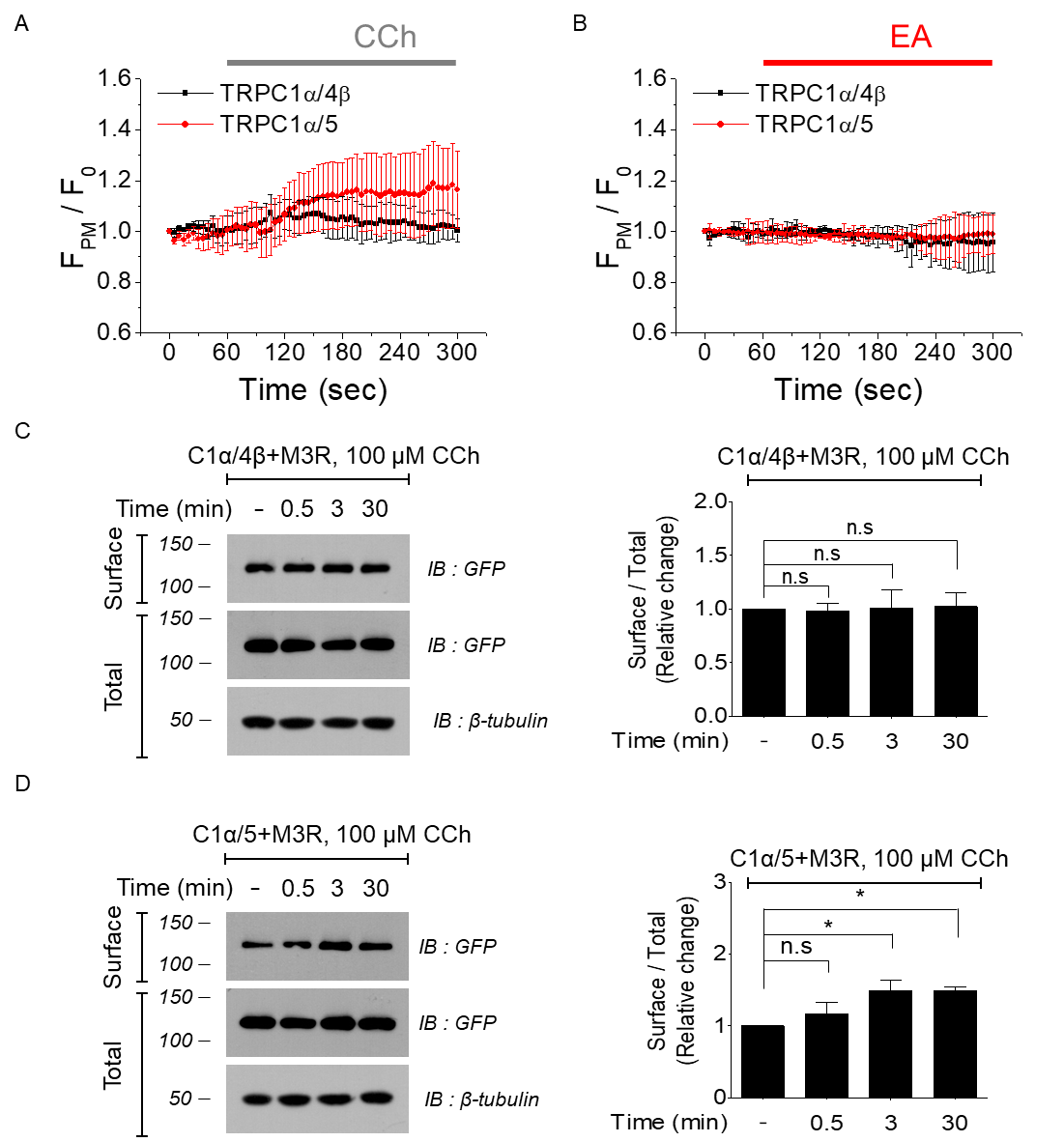


**Supplementary Figure 11. Membrane expression change by CCh or EA stimulation**

Cells expressing TRPC1α/4β or TRPC1α/5 channels were observed using a TIRF microscope after stimulation with (A) 100 μM CCh or (B) 100 nM EA. When 100 μM CCh stimulation was used in the surface biotinylation experiment, the changed in membrane expression of (C) TRPC1α/4β and (D) TRPC1α/5 channels was observed over time. All data are presented as the mean ± SEM. n.s., not significant. **p <* 0.05.


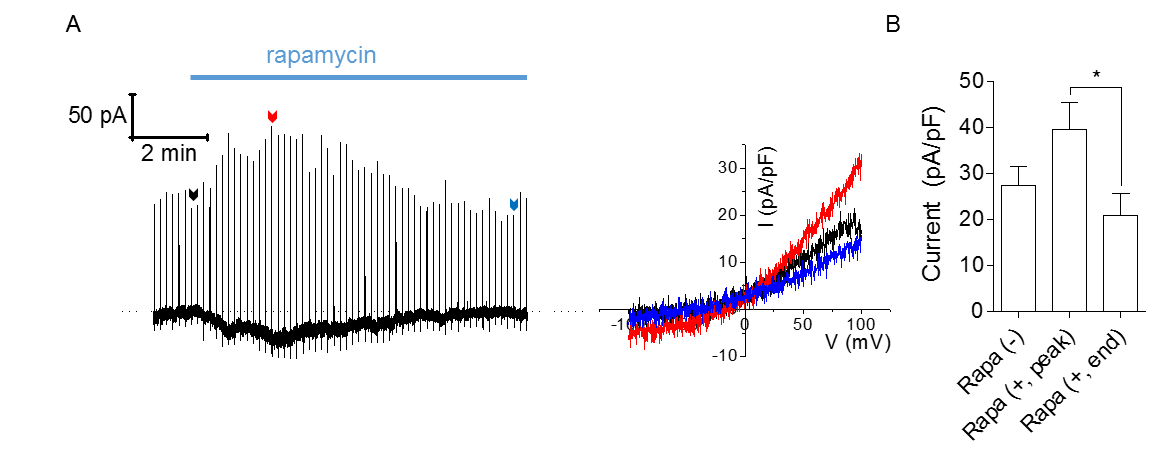


**Supplementary Figure 12. Transient activation of TRPC1α/4β current by Gα_q_ and PI(4,5)P_2_ depletion**

(A) TRPC4β, YFP-TRPC1α, Inp54-FKBP, RFP-FKBP-Gα_q_ (Q209L, L254A), and Lyn-FRB were coexpressed in HEK293 cells, Rapamycin-induced Full traces and I/V curves are presented. (B) Basal, peak, and desensitization current of TRPC1α/4β with rapamycin at +100 mV. All data are presented as the mean ± SEM; n.s., not significant. *p < 0.05.
